# Exogenous treatment of Arabidopsis seedlings with lyso-phospholipids for the inducible complementation of lipid mutants

**DOI:** 10.1016/j.xpro.2021.100626

**Published:** 2021-06-23

**Authors:** Matthieu Pierre Platre, Yvon Jaillais

**Affiliations:** 1 Plant Biology Laboratory, Salk Institute for Biological Studies, 10010 N Torrey Pines Rd, La Jolla, CA 92037, USA; 2 Laboratoire Reproduction et Développement des Plantes, Université de Lyon, ENS de Lyon, UCB Lyon 1, CNRS, INRAE, 69342 Lyon, France

**Keywords:** Cell Membrane, Microscopy, Model Organisms, Plant sciences, Molecular/Chemical Probes

## Abstract

Lipids are major components of membranes with pleiotropic roles and interconnected metabolism, so experimentally addressing the primary function of individual lipid species *in vivo* can be difficult. Genetic approaches are particularly challenging to interpret due to compensatory mechanisms and indirect effects. Here, we describe a fast inducible approach to complement the phenotypes of *Arabidopsis* lipid mutants through exogenous treatment with the depleted lipid, followed by live confocal imaging to observe genetically encoded lipid sensors in wild-type and mutant root tissues.

For complete details on the use and execution of this protocol, please refer to Platre et al. (2018).

## Highlights

• Protocol for the inducible complementation of lipid mutants using lyso-phospholipids
• Live imaging of root tissue by confocal microscopy
• Observation of genetically encoded lipid sensors in wild-type and mutant background
• Protocol for the quantitative analysis of rosette size

## Graphical abstract

**Figure:**
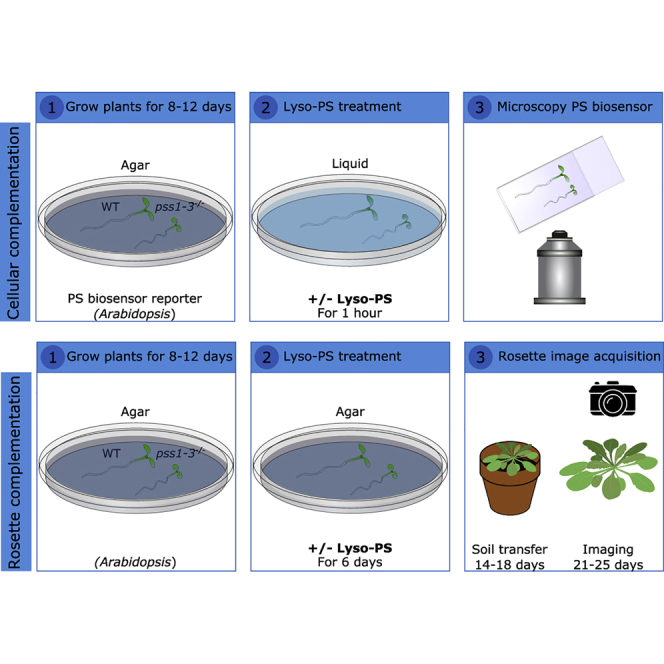

## Before you begin

Here, we give examples on phenotypical complementation at the macroscopic and cellular levels of *Arabidopsis phosphatidylserine synthase 1* (*pss1*) mutant plants using lyso-phosphatidylserine (lyso-PS). At the macroscopic level, we compare the size of the *pss1* mutant rosette with that of the wild type in the presence or absence of lyso-PS. At the cellular level, we use transgenic wild type and *pss1* mutant seedlings stably expressing a genetically encoded *in vivo* PS biosensor. This biosensor is based on the stereospecific PS-binding pleckstrin homology (PH) domain of the human protein EVECTIN 2 (EVCT2)(Platre and Jaillais, 2016; Uchida et al., 2011). It is arranged as a tandem dimer to provide high affinity binding, fused to the yellow fluorescent protein mCITRINE for visualization and expressed under the control of the ubiquitous promotor of the *UBIQUITIN10* gene (UBQ10prom::mCITRINE-2xPH^EVCT2^; Platre et al., 2018). This PS biosensor is a soluble fusion protein synthesized in the cytosol. It is subsequently targeted to the cytosolic membrane leaflets of certain endomembrane compartments thanks to its specific interaction with PS (Platre and Jaillais, 2016). As such, it is localized at the plasma membrane and as a gradient along the endocytic pathway in the wild type (Dubois and Jaillais, 2021; Platre et al., 2018). However, in the absence of PS in the *pss1* mutant background, this sensor is localized by default in the cytosol (i.e., it is not associated with any membrane anymore because of the lack of PS in this mutant) (Platre et al., 2018). It is thus possible to use the localization of mCITRINE-2xPH^EVCT2^ (at the plasma membrane/endosomes v.s. in the cytosol) as a cellular read-out to test whether the exogenous treatment of the *pss1* mutant with lyso-PS induces the presence of PS in the cytosolic membrane leaflet of this mutant. We also used lyso-phosphatidic acid (lyso-PA) to show the specificity of the lyso-PS treatment for the inducible complementation of *pss1*. It should be noted that the same protocol can be developed with any other readouts using different lipid mutants (or chemical inhibition of lipid metabolism), lipids and their related lipid biosensors. For example, we used exogenous lyso-PA treatment to complement protein localization phenotypes induced by the chemical inhibition of diacylglycerol kinases, which inhibits phosphatidic acid synthesis at the plasma membrane (Platre et al., 2018). In that case, we used a PA-specific lipid biosensor to control the effect of 1) the chemical inhibition of diacylglycerol kinases and 2) the complementation induced by lyso-PA (Platre et al., 2018).

### Preparation of Arabidopsis seedlings

**Timing: 12 days**

1. Prepare ½ MS agar plates for *Arabidopsis* seedlings
  a. Dissolve 2.2 g/L Murashige & Skoog (MS) Basal Medium (without vitamins) and 1 g/L of sucrose, fill up to 1L with ddH_2_O. Add 8 g/L of Agar and adjust pH to 5.7 with 1 M KOH. Autoclave and place it in a water bath at 55°C for at least 3 h before use. This is highly recommended to use fresh media.

**Table:** 

Reagent	Final concentration	Amount
Murashige & Skoog Basal Medium	2.2 g/L	2.2 g
Sucrose	1 g/L	1 g
Agar	8 g/L	8 g
ddH_2_O	n/a	fill up to 1 L
  b. In a sterile hood pour 50 mL in 12 × 12 mm plates.
  c. Wait for 30 min with lid open to let the media dry and excess humidity evaporate.
  d. Close the plates.

***Note:*** It is better to use the plates poured on the same day, otherwise it is possible to store them in a sealed bag at 4°C for a week. Moreover, in order to identify easily *pss1-3*^*-/-*^ mutant root phenotypes this is recommended to use sucrose in the media as indicated step 1.a.

2. Sterilize seeds
  a. Aliquot the seeds of *Arabidopsis* genotypes, wild type (WT), self-progeny of *pss1-3*^*+/-*^, WT plants and self-progeny of *pss1-3*^*+/-*^ expressing the *in vivo* PS biosensor line (*UBQ10::mCITRINE-2xPH*^*EVCT2*^) in 1.5 mL Eppendorf tubes. For efficient sterilization, do not fill up the tube with seeds more than 2 millimeter from the bottom which represents about 100 μL of seeds. Annotate the tubes with permanent waterproof pen to make sure the labels are still visible after sterilization. Certain marker or color may be erased by the sterilization treatment, we thus advice to first test it is not the case before sterilizing several tubes in parallel.
  b. Do not close the lid of the Eppendorf tubes.
  c. Place the tubes on a tube rack in an airtight plastic box and place it in a fume hood.
  d. In a 500 mL glass beaker pour 200 mL of commercial bleach and place it in the plastic box.
  e. Pour 3.5 mL of hydrochloric acid into the beaker.
  f. Close the plasticware box.
  g. Wait an hour.
  h. Open the lid and remove the glass beaker from the box.
  i. Let ventilate for 30 min.
  j. Close the lid and move the plastic box to a sterile hood.
  k. Remove the tube rack from the plastic box and leave it under the sterile hood with Eppendorf tubes open.

***Optional:*** You can also close the Eppendorf tubes and use the seeds later.

***Note:*** The *pss1-3*^*-/-*^ mutant can grow for more than 3 months in standard condition but is sterile and thus can be propagated only as a heterozygous. To identify homozygous *pss1-3*^*-/-*^ mutant in a segregating population of seedlings coming from self-fertilized *pss1-3*^*+/-*^, the seedlings are grown for at least 8 to 12 days after stratification on vertical plates and recognized based on their agravitropic root phenotypes (Platre et al., 2018, 2019).

**CRITICAL:** Mixing bleach with hydrochloric acid will produce chlorine gas, which is used for sterilization purposes here but is also very harmful if inhaled. It is imperative to set-up this reaction under a proper fume hood (not just a sterile hood) and to wear gloves and protection glasses when handlings these chemicals.

3. Seeds sowing
  a. In a sterile hood, spread the sterilized seeds on the plate containing ½ MS agar growing media.
  b. Close the lid and seal the plate with micropore surgical tape.
  c. Wrap the plates in an aluminum foil and transfer them at 4°C for 2–3 days.
  d. Remove the aluminum foil and place the plates vertically in a growth chamber in continuous light conditions 150 μE.m^−2^.s^−1^ at 21°C for 8–12 days.

### Preparation of lyso-PS stock solution

**Timing: 1 h**

4. Prepare stock solution of lyso-PS (or lyso-PA for control)
  a. Take the vial of C18:1 lyso-PS (or C18:1 lyso-PA) dissolved in chloroform at 10 mg/mL (stored at −20°C for a maximum of a year).
  b. Using a 25 μL Hamilton syringe, transfer 12 μL of stock lyso-PS chloroform solution or 10 μL for lyso-PA to a 1 mL shell vial to prepare the solutions at steps 5c and 6b with a final concentration of about 219 μM.
  c. Dry off chloroform by an argon gas flux until it makes a film at the bottom of the vial and that indicates all chloroform has evaporated.
  d. Saturates the shell vial with argon, close the lid and wrap it with parafilm.

***Note:*** We usually prepare several vials at once to minimize opening the stock solution of chloroform / lyso-PS. These aliquots may be used immediately or within a month. The remaining stock solution can be closed and sealed with parafilm to be stored at −20°C for a maximum of a year.

**CRITICAL:** To avoid lipid oxidation, the lipid solution should be in an argon saturated atmosphere at all time. For this, always manipulate your samples under a chemical hood and place a continuous argon flux over your open vials. After used, you should also make sure the shell vial containing the dried lipid and the lyso-PS chloroform stock solution are saturated with argon gas.

***Optional:*** The argon gas is used because it is an inert gas thereby limiting lipid oxidation. It is equally possible to use another inert gas such as Nitrogen.

### Preparation of liquid and agar media with lyso-PS

**Timing: 4 h**

5. Prepare ½ MS liquid media with lyso-PS (or lyso-PA)
  a. Add 1.1 g of MS Basal medium (without vitamins) and fill up with 500 mL of ddH_2_O. Adjust pH to 5.7 with 1 M KOH and autoclave. Cool it down at room temperature (RT, about 20°C). This media is named ½ MS liquid media.
  b. In a sterile hood, aliquot ½ MS in 50 mL Falcon tubes (stored at 4°C for maximum a month). If stored at 4°C, warm up the media at RT for 1 h before adding lyso-PS stock.
  c. In a sterile hood, add 1 mL of liquid ½ MS in the shell vial containing 12 μL of lyso-PS (as prepared in step 4) to get a solution at 219 μM, close and vortex for 10 s. This step allows to resuspend the lyso-PS.
  d. Do a 4× dilution (taking the 0.5 mL and adding 1.5 mL of ½ MS liquid media) to get a final solution at 54 μM. This solution has to be poured in one of the well of the 12 wells plates and should be used right away.

***Note:*** In the original paper, liquid ½ MS media was supplemented with BSA. Later experiments confirmed that BSA does not need to be added and subsequently has been removed from the protocol.

6. Prepare plates of ½ MS agar media with lyso-PS (or lyso-PA)
  a. Dissolve 1.1 g/L Murashige & Skoog (MS) Basal Medium (without vitamins) and fill up to 500 mL with ddH_2_O. Add 4 g/L of Agar and adjust pH to 5.7 with 1 M KOH. Autoclave and place it in a water bath at 55°C for at least 3 h before use. This media is named ½ MS agar media. This is highly recommended to use fresh media.

**Table:** 

Reagent	Final concentration	Amount
Murashige & Skoog Basal Medium	2.2 g/L	1.1 g
Agar	8 g/L	4 g
ddH_2_O	**n/a**	fill up to 500 mL
  b. In a sterile hood, add 1 mL of ½ MS liquid media in the shell vial containing 12 μL of lyso-PS (as prepared in step 4) to get a solution at 219 μM, close and vortex for 10 s. This step allows to resuspend the lyso-PS. Repeat this step 5 times total to obtain 5 mL of lyso-PS solution at 219 μM.
  c. Mix 5 mL of lyso-PS solution made in step 6b with 500 mL of ½ MS agar media to reach a final concentration of about 2.19 μM lyso-PS and pour the plates.

***Note:*** Use the plates the same day they have been poured.

***Optional:*** The final lysophospholipid concentration can be adapted according to the experiments. However, empirical tests will be required when increasing the lipid concentration in order to control for lipid solubility and the potential effect on the agar medium. Indeed, high lipid concentration may alter the agar property and make it less stable when positioned vertically. Note that we have not tried higher lyso-PS concentration in MS agar plates mainly because of the high cost of such experiments.

### Preparation of growing soil for Arabidopsis

**Timing: 45 min**

7. Prepare soil
  a. Fill up the pots with soil and press down to compact the soil
  b. Place the pots into a tray
  c. Fill up the tray with H_2_O and wait for soaking at least for 30 min

## Key resources table

**Table:** 

REAGENT or RESOURCE	SOURCE	IDENTIFIER
**Chemicals, peptides, and recombinant proteins**		

Lyso Phosphatidylserine 18:1	Avanti Polar Lipids	Cat #858143
Lyso Phosphatidic acid 18:1	Avanti Polar Lipids	Cat #857130
Agar	Sigma-Aldrich	Cat #V900500
Murashige and Skoog (MS) Basal Medium (without vitamins)	Sigma-Aldrich	Cat #M5519
KOH	Sigma-Aldrich	Cat #P5958
Sucrose	Sigma-Aldrich	Cat #S0389
HCL	Sigma-Aldrich	Cat #H1758
Bleach	Clorox	Cat #30966

**Experimental models: Organisms/strains**		

*Arabidopsis thaliana*: Col-0 WT	Nottingham Arabidopsis Stock Center	NASC #N1092
*Arabidopsis thaliana*: *pss1-3*^*-/-*^	(Platre et al., 2018)	NASC #415992 GABI_166G10
*Arabidopsis thaliana*: *pUBQ10::mCITRINE-2xPH*^*EVCT2*^	(Platre et al., 2018)	NASC #N2107779
*Arabidopsis thaliana*: *pUBQ10::mCITRINE-2xPH*^*EVCT2*^ in *pss1-3*^*-/-*^	(Platre et al., 2018)	N/A

**Software and algorithms**		

Fiji	(Schindelin et al., 2012)	https://fiji.sc/
Excel	Microsoft	https://www.microsoft.com/en-us/microsoft-365/excel
Xlstat	Microsoft	http://www.xlstat.com/.
Macro_Rosette_Size	This study	https://github.com/mplatre/Macro_Rosette_Size/releases/tag/V1

**Other**		

Plates, petri dish 12 × 12 mm	Sigma-Aldrich	Cat #Z692344
12-Well plates	Sigma-Aldrich	Cat #CLS3737
Micropore Surgical Tape	3M	Cat #6510008901369
Shell vial	Fisher Scientific	Cat #03-375-2G
Hamilton syringe	Sigma-Aldrich	Cat #24531
50 mL Falcon tubes	Fisher Scientific	Cat #14-432-22
1.5 mL Eppendorf tubes	Sigma-Aldrich	Cat #EP022364120
Tube rack	Sigma-Aldrich	Cat #EP0030119819
Microscope Slides	Fisher Scientific	Cat #12-550-123
Cover slips	Fisher Scientific	Cat #50143697
Waterproof pen	Fisher Scientific	Cat #50-809-275
Argon line	N/A	N/A
Autoclave	N/A	N/A
Water bath	N/A	N/A
Fume hood	N/A	N/A
Sterile hood	N/A	N/A
Growth chamber	N/A	N/A
Tweezers	N/A	N/A
Macroscopic Camera Canon EOS 450D with a Sigma DC 18–50 mm 1:2.8 EX Macro lens	(Platre et al., 2018)	N/A
Assembled Spinning Disc, with inverted Zeiss microscope AxioObserver Z1, Carl Zeiss Group, equipped with a spinning disk module (CSU-W1-T3) and a ProEM+ 1024B camera and a 63× Plan-Apochromat objective (numerical aperture 1.4, immersion oil)	(Platre et al., 2018)	N/A
Immersion oil	Weber Scientific	Cat #2033-00
Plastic pots	Growers House	Cat #724042-0
Trays	Growers House	Cat #726298
Soil with fertilizers	Miracle-Gro	Cat #75686300

## Materials and equipment

The key resources table details all required materials and equipment.

***Alternatives:*** Any plant growth media, seed sterilization methods, soil, confocal microscope, camera and software can be used instead of the one abovementioned.

## Step-by-step method details

### Complementation at the cellular level by lyso-PS

**Timing: 3 h**

To complement PS-deficient plants at the subcellular level, we studied the subcellular localization of a PS biosensor in WT and *pss1-3*^*-/-*^ seedlings adding back external lyso-PS. Briefly, WT and *pss1-3*^*-/-*^ mutant seedlings expressing the PS biosensor (*pUBQ10::mCITRINE-2xPH*^*EVCT2*^) are treated in ½ MS liquid media supplemented or not with lyso-PS (or lyso-PA as control) and then images are acquired using a confocal microscope.

**Figure 1:**
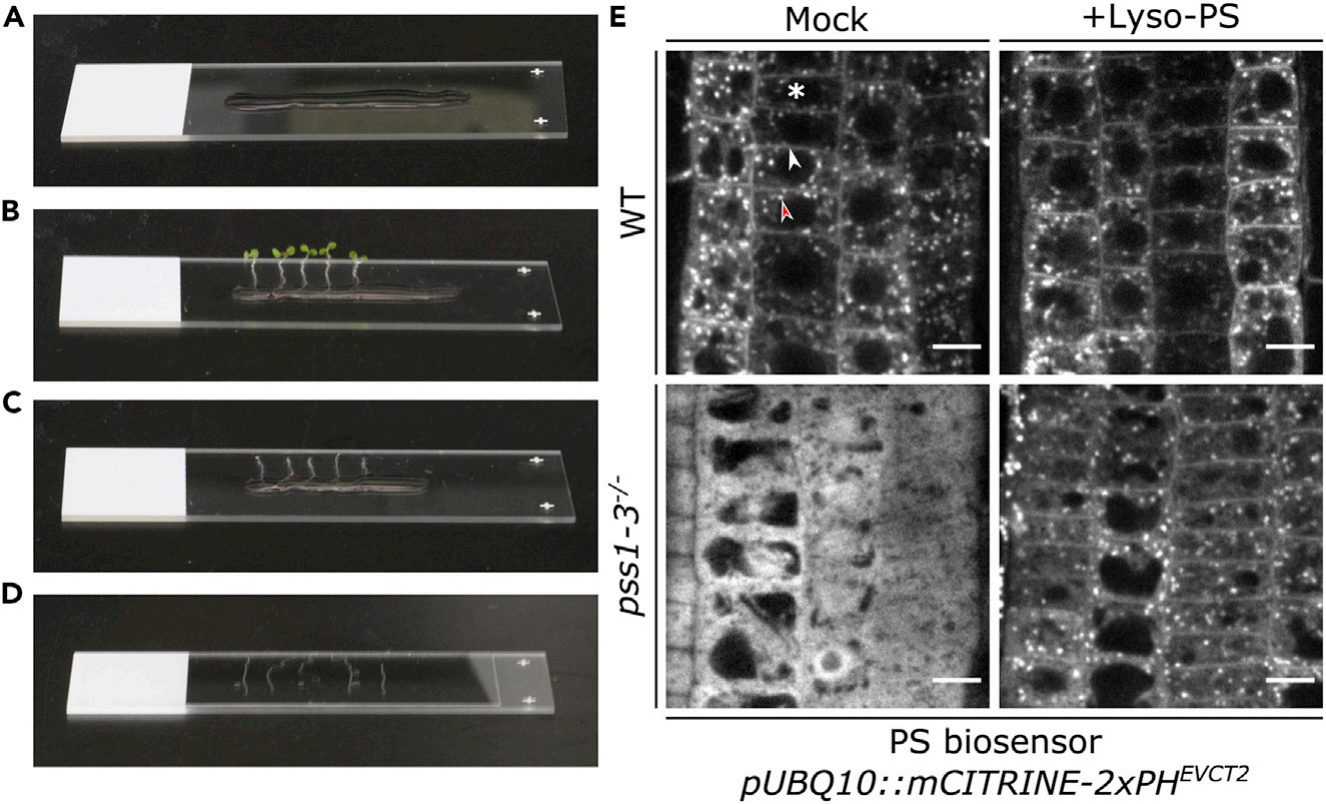
Complementation of PS biosensor localization in *pss1* using lyso-PS (A–D) Images of microscope slides with ½ MS (with or without lyso-PS) (A), ½ MS (with or without lyso-PS) and seedlings (B), ½ MS (with or without lyso-PS) and decapitated seedlings (C) and ½ MS (with or without lyso-PS), decapitated plants and cover slip. (E) Confocal images of 8–12 days old seedling expressing PS biosensor (*UBQ10::mCITRINE-2xPH*^*EVCT2*^) in WT (upper panels) and in *pss1-3*^*-/-*^ (lower panels) under mock condition (left panels) and under lyso-PS treatment for an hour (right panels). Asterix indicates cell which shows larger width than length, white arrow indicates plasma membrane and red arrow indicates endosomes. Scale bars 5 μm.
  b. Place about 5 seedlings in the drop on the microscope slide and orient the root perpendicular to the main axis of the slide (Figure 1B).
  c. Remove the shoot with a scalpel (Figure 1C).

1. Treat seedlings with lyso-PS
  a. Prepare 12-wells plates containing 2 mL of ½ MS liquid media supplemented or not with lyso-PS at 54 μM (as prepared in step 5).
  b. Place about 10–15 of 8 to 12-day-old seedlings of each genotype in wells supplemented with or without lyso-PS. Make sure that the seedlings are drowned in the media.
  c. Transfer the plate in a growth chamber in continuous light conditions 150 μE.m^−2^.s^−1^ at 21°C for an hour.
2. Prepare microscope
  a. Use an inverted Zeiss microscope equipped with a spinning disk module and a ProEM+ 1024B camera using a 63× Plan- Apochromat objective
  b. Turn on the 515 nm laser for mCITRINE excitation (60 mW)
  c. Set fluorescence emission filter to 578/105 nm, using BrightLine® single-band bandpass filter (Semrock, http://www.semrock.com/).
  d. Clean the objective before usage applying 70% ethanol using a lens paper.
3. Mount seedlings on microscope slides
  a. When using 60 millimeters wide cover slips, add about 75 μL of corresponding liquid ½ MS with or without lyso-PS in the middle of the slide with a plastic Pasteur pipette. If 40 millimeters cover slips are used, add about 50 μL instead (Figure 1A).

Place the cover slip gently on the sample to avoid crushing the root (Figure 1D).

***Note:*** After placing the coverslip on the samples, if the entire coverslip is not fully covered with liquid ½ MS, add-back liquid ½ MS in the interstice between the slide and the coverslip using a plastic Pasteur pipette. If too much water has been added, the cover slip might move around. In that case, place a cleaning tissue in the interstice between the slide and the coverslip to absorb excessive water. It is also possible to tape the slide and the coverslip together with surgical tape to avoid movement of the coverslip (Bayle et al., 2021).

**CRITICAL:** Prior to placing the plants on the slide, make sure that the roots have been dipped in the in the corresponding liquid ½ MS to avoid the seedlings from drying.

***Optional:*** Removing the shoot is optional as for certain experiments, it might stress the plants. It is thus possible to mount the entire seedlings, without removing the shoot, by simply placing the shoot, starting from the base of the hypocotyl, outside of the slide/coverslip sandwich (Figure 1B). To avoid crushing the samples, it is also possible to make spacers between the slide and the coverslip. To do that, put two to three layers of surgical tape around the slide, on both sides of the samples. Make both spacers of equal thickness on both side of the samples to ensure that the slide and the coverslip remains parallel (Bayle et al., 2021).

4. Imaging of PS biosensor
  a. Pour one drop of immersion oil on the objective
  b. Place the slide on the microscope stage.
  c. Using the brightfield to find the root tip.
  d. Adjust the x-y-z position to observe the epidermis in the basal meristem.
  e. Acquire images with settings allowing to obtain the best signal/noise ratio and to avoid signal saturation. This can be monitored by observing the diagram of the image dynamic or using the range indicator (Figure 1E).

***Note:*** To find the epidermis in the basal meristem, make sure that the focus is adjusted on cells which correspond to the outer layer of the root and with a width larger than the length as indicated in Figure 1E with the asterix. The epidermis can easily be distinguished from the lateral root cap, because epidermal cells in the meristem are not fully elongated and differentiated (they have small vacuoles), while cells in the lateral root cap are elongated and have a big central vacuole.

**CRITICAL:** Make sure that the imaged root has not been damaged during the slide mounting process by visualizing rectangle cells with a nearly straight plasma membrane as in Figure 1E.

***Optional:*** We image the meristematic zone since meristematic cells have small vacuoles and an expanded cytosol, making the comparison between soluble and membrane-localized mCITRINE-2xPH^EVCT2^ easier. However, the difference of localization of this PS sensor between the wild-type and *pss1* mutant can be easily distinguished in all the cell types that we have looked at, including in the root meristem, differentiated cells of the root and shoot tissues. Thus, it is possible to analyze the localization of the PS sensor in a wide range of cell types, not only root meristem epidermis, to check whether the lyso-PS treatment was efficient.

5. Image analysis
  a. Evaluating the subcellular localization of the PS biosensor by eyes is sufficient. No quantification is needed (Figure 1E). Evaluate the phenotype based on the criteria listed below:
    i. The PS biosensor is localized at the plasma membrane and endosomes in the WT which looks like thin lines around the cell and dots inside the cells, respectively.
    ii. The PS biosensor is localized in the cytosol in *pss1-3*^*-/-*^ mutant which looks like a spread and smooth signal inside the cell with black round holes corresponding to the nucleus and vacuoles.
    iii. The PS biosensor is localized at the plasma membrane and endosomes in *pss1-3*^*-/-*^ treated with lyso-PS mimicking the localization in the WT

### Complementation at the macroscopic level by lyso-PS

**Timing: 21 days**

In order to observe complementation of *pss1-3*^*-/-*^ at the macroscopic level using lyso-PS, we used root growth and the size of the rosette as a readout. Briefly, WT and *pss1-3*^*-/-*^ mutant seedlings are first grown for 8–12 days in ½ MS agar plates. They are then dipped in ½ MS liquid media supplemented or not with lyso-PS and transferred to ½ MS agar media supplemented or not with lyso-PS for 6 days. Finally, seedlings are transferred in soil for 8 days before image acquisition for quantification.

**Figure 2:**
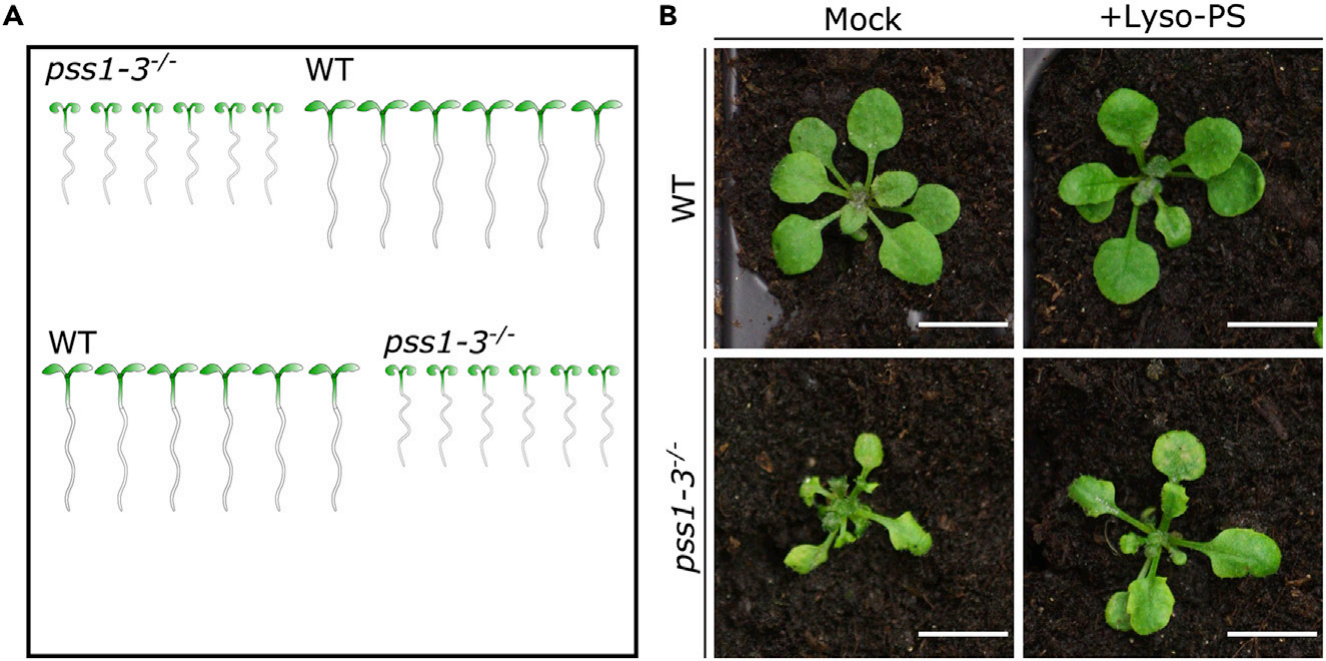
Complementation of *pss1-3*^*-/-*^ rosette phenotype using lyso-PS (A) Scheme of genotypes organization in 12 × 12 mm petri dish with or without lyso-PS. (B) Images of rosette of 21–25 days old plant in WT (upper panels) and *pss1-3*^*-/-*^ (lower panels) under mock condition (left panels) and under lyso-PS treatment for 6 days and then transfer for 8 days in soil (right panels). Scale bars 1 cm.
  b. Seal the plates with micropore surgical tape.
  c. Place the plates vertically in a growth chamber in continuous light conditions 150 μE.m^−2^.s^−1^ at 21°C for 6 days.

***Note:*** To sterilize the tweezers, dip them in ethanol 70% and flame dry them by quickly passing the ethanol-soaked tweezers into the flame (using a Bunsen burner or an alcohol burner). Make sure that the tweezers are cooled down before starting to transfer the plants in order to avoid burning and stressing the tissue.

6. Transfer seedlings to ½ MS agar plates
  a. In a sterile hood, transfer 6 seedlings of 8–12 days old alternating sides on plate supplemented or not with lyso-PS using sterile tweezers. (Figure 2A)

***Optional:*** Before transfer, dip the plants in the corresponding liquid media for 5 s. While the plants are growing, the plates can be scanned using EPSON scanner perfection V300 PHOTO at 800 dpi to measure the root growth rate as described in Platre et al., 2018.

7. Transfer the seedlings in soil
  a. Using tweezers, transfer two seedlings per pot.
  b. Use a tag indicating the genotype and the media in which the seedlings have been growing in.
  c. Grow the plants in a growth chamber in long day (18 h light, 6 h dark) light conditions 150 μE.m^−2^.s^−1^ at 21°C for 8 days.

**CRITICAL:** Make sure that the plants are not too close from each other when transplanting them, and in addition, ensure that plants are away from the pot edges to allow proper quantification**.**

***Note:*** In our condition, 8-day-old plants are enough to observe significantly complemented rosette phenotype in *pss1-3*^*-/-*^ by eyes. To avoid the effect of overlapping rosette in WT plants, proper quantification is required. However, any time before 8 days can be used.

8. Image acquisition of the rosette
  a. Using a camera and a macro objective, in our case (CANON EOS 450D with a SIGMA DC 18–50 mm 1:2.8 EX MACRO lens) to take pictures (Figure 2B).
  b. Use setting to optimize the color between the rosette and the background, automatic settings should be sufficient.
  c. In one of the picture place a ruler to be able to update the image scale in centimeters (cm) for quantification

**CRITICAL:** It is imperative to use a camera holder to make sure the distance between the objective and the plants is staying the same between the different images.

**Figure 3:**
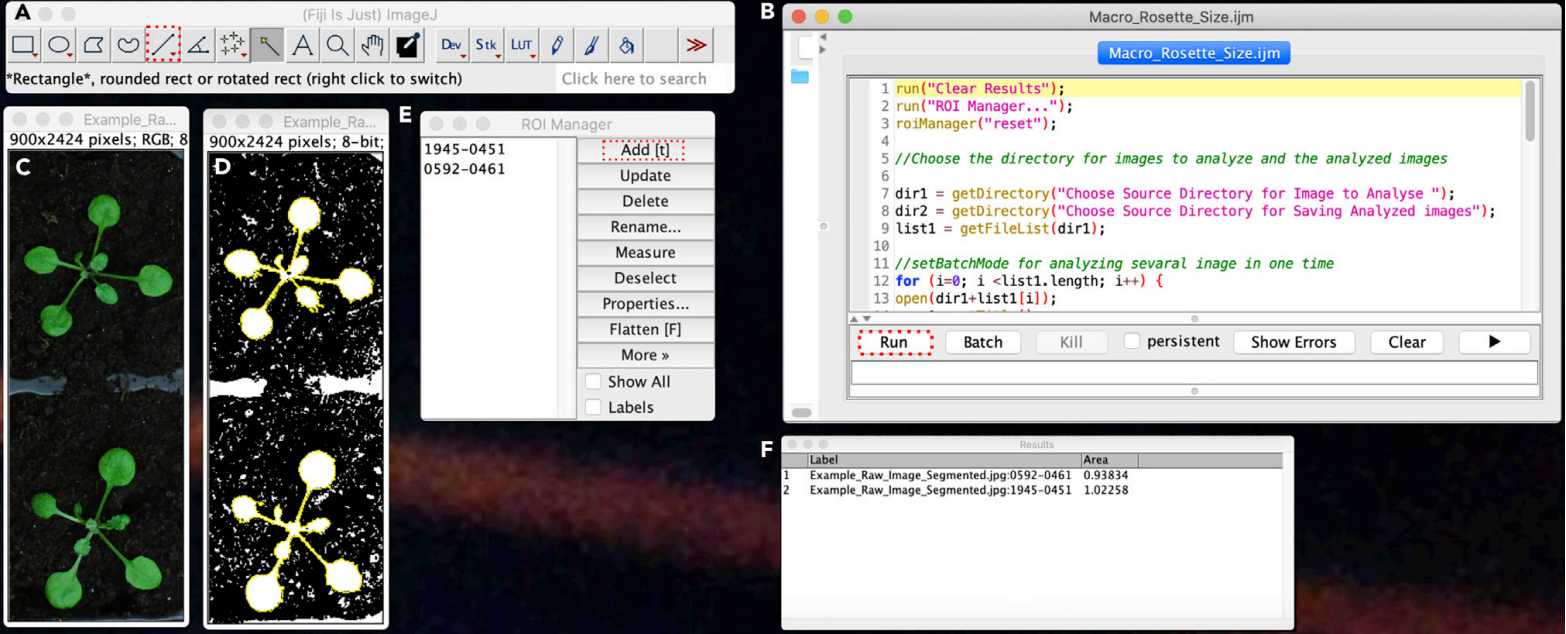
Measuring rosette size on Fiji (A) The Fiji tool bar with the “Straight Line” button highlighted by the red dashed line. (B) The ROI manager with the “Add [t]” button highlighted by the red dashed line. (C) Raw images of the rosette. (D) Segmented rosette by the “Macro_Rosette_Size”. (E and F) (E) Macro_Rosette_Size window with the “run” button highlighted by the red dashed line (F) Results table showing the area of the rosette expressed in cm square.
    iii. Click on Analyze/Set Scale…
    iv. In the box “Known distance” write “1” and in “Unit of length” write “cm” and activate the “global” mode. And click on OK.
  e. Open the “Macro_Rosette_Size.ijm” in Fiji, dragging the macro “Macro_Rosette_Size.ijm” file in the tool bar (Figure 3B).
  f. Click on “Run” (Figure 3B).
  g. Select the folder “To Analyze_Genotype_Condition” and click OK for your input images. Next, select the folder “Analyzed_Genotype_Condition” to save Macro-processed images.
  h. The image will be then processed to segment the rosette (Figures 3C and 3D). Click on the rosette 1. If the rosette has been separated in different parts during the image processing, press the shift button on the keyboard and click on the separated part. Click on Add [t] in the ROI Manager window. (Figure 3E). Repeat this step for the rosette 2. And click on OK
  i. After analyzing all the image, you should obtain a window named “results” containing the size in cm^2^ for each rosette measured (Figure 3F).

9. Image quantification (Figure 2)
  a. For each condition and genotype, store the image to be analyzed in a separated folder named “To Analyze_Genotype_Condition” and create a new empty folder named “Analyzed_Genotype_Condition”
  b. Use Fiji to open images
  c. Set Fiji
    i. Click on Analyze/Set Measurements…
    ii. Activate “Area box” and click on OK
  d. Set the scale
    i. Open the image containing the ruler on Fiji
    ii. Use the straight-line tool and trace 1 cm on the ruler (Figure 3A).

## Expected outcomes

### Complementation at the cellular level by lyso-PS

The localization of the PS biosensor can be visualized from the confocal pictures acquired in the root epidermis of the WT and *pss1-3*^*-/-*^ mutant in treated or non-treated conditions with lyso-PS (Figure 1E). In WT plants without lyso-PS add-back, the PS biosensor is mainly associated with the plasma membrane and intracellular compartments such as trans-golgi network/early endosomes, golgi, late endosomes (Platre et al., 2018). This particular localization pattern is observed by a continuous faint signal surrounding the cell corresponding to the plasma membrane (as shown in Figure 1E by the white arrow) and by the intracellular doty signal corresponding to endosomes (as shown in figure 1E by the red arrow). In *pss1-3*^*-/-*^ mutant background, the signal of the PS biosensor is localized in the cytosol which looks like a spread and smooth signal inside the cell with black round holes corresponding to the nucleus and vacuoles (Figure 1E). The add-back of lyso-PS for one hour in WT does not affect the PS biosensor; however, we sometimes noticed a stronger signal associated with the plasma membrane. In *pss1-3*^*-/-*^ mutant background, this experiment leads to a relocalization of the PS biosensor at the plasma membrane and endosomes as observed in the WT albeit a remaining fraction can still be cytosolic.

### Complementation at the macroscopic level by lyso-PS

The size of the rosette can be visualized in the images acquired with the camera (Figure 2B). The area of *pss1-3*^*-/-*^ is about 6 time smaller than the WT in the absence of lyso-PS while adding the lyso-PS the area in about 3 time smaller than the WT (Figure 2B).

## Quantification and statistical analysis

For the analysis of the subcellular localization of the PS biosensor no quantification and statistical analysis need to be performed. Evaluation by eyes is sufficient since the difference is very drastic between WT and *pss1-3*^*-/-*^ in treated or non-treated conditions with Lyso-PS (Figure 1E). If quantification is required, it is possible to use the dissociation index as introduced in Simon et al., 2016 or to automatically count the numbers of labeled intracellular compartments (Bayle et al., 2017).

For complementation of the rosette phenotype, copy the “Results” table (Figure 3F) appearing in Fiji after measurement and past the data in an Excel sheet. Each value should be referenced with the date, experience number, genotype, treatment, Number, Label and Area (Table 1). Use the pivot table to organize the data as you wish for further statistical analysis, using “Insert/Pivot Table”. Then using Xlstat, perform a normality test clicking on “Describing data/Normality test” and perform all the test available. If the test indicates that the sample follows a normal distribution apply a two ways student test (p=0.05) using “Parametric test/two sample t-test and z-test” or if it does not apply a Mann-Whitney test (p=0.05) using “non-parametric test/comparison of two samples”.

**Table 1:** Example of the rosette size raw data measured with Fiji and reported in Excel for statistical analysis

Genotype/condition	Average of area (cm^2^)	Percentage of WT (%)
**WT**		
Mock	1.03	100
lyso-PS	1.06	100

***pss1-3***^***-/-***^		

Mock	0.17	16
lyso-PS	0.34	33

## Limitations

It is possible that the exogenously added lipid is quickly metabolized into another lipid species. This can be checked using TLC, lipidomic, and/or specific lipid biosensors when available. If this is the case, care must be taken when interpreting the resulting cellular and developmental phenotypes.

Exogenously added lipids may not accumulate in the same membranes in which they would endogenously accumulate. This is a well-known limitation of this approach. In particular, it is sometime not possible to compare the subcellular accumulation of the exogenously added lipid with its endogenous localization, notably when the corresponding lipid biosensors are not available (D’Ambrosio et al., 2019). In such case, care must be taken when interpreting data coming from lipid addback experiments taking into account this possible limitation. Moreover, this difference can be explained by the time frame at which the biosensor subcellular localization is observed after the lipid addback as observed in yeast (D’Ambrosio et al., 2019).

While we know the concentration of the lipid that we apply, it is impossible to know what is the amount of exogenous lipids that are incorporated in the cells.

It is possible that different cell types may receive different amount of exogenous lipids, which can ultimately impact the plant phenotype.

## Troubleshooting

### Problem 1

No complementation of the subcellular localization of the PS biosensor or the rosette phenotype of the *pss1* mutant (steps 5 and 2). This problem might occur if: i) the lyso-PS preparation from the chloroform has been stored for more than a year in the freezer at −20°C, ii) the lyso-PS preparation from the chloroform stock solution has not been perform properly (e.g., trace of chloroform, time to evaporate the chloroform with argon has been too long and the lipids have been oxidized), or iii) the lyso-PS in the argon has been stored for more than a month in −20°C.

### Potential solution

To solve the problem, prepare a fresh stock of lyso-PS when performing complementation experiments.

### Problem 2

No phenotype observed when testing the effect of the exogenous treatment with a new phospholipid or lyso-phospholipid (steps 5 and 2). This problem might occur if i) the lipid is not well solubilized at this concentration, or ii) it is not incorporated into the endomembrane system (blocked by the cell wall, not able to flip/flop into the cytoplasmic membrane leaflet for example).

However, it should be noted that it is fine to observe no phenotype if there is not an expectation of phenotype. For example, lyso-PA is used as a control of the *pss1* rescue experiment (Platre et al., 2018).

### Potential solution

If available, use a lipid sensor to check the effect at the cellular levels of lipid add-back experiments and try different time period after the lipid add-back. If no effect can be seen at the developmental and cellular levels, try different lipid concentration (by setting-up a dosage response curve). Try also different lipid molecular species with different acyl chain length and saturation degree.

### Problem 3

When doing add-back experiments on plates, the plates are greasy (step 6). This problem might occur if the lipid concentration is too high.

### Potential solution

Decrease the lipid concentration. If a higher lipid concentration is required, keep the plate horizontal to avoid sliding of the greasy agar.

### Problem 4

Limited complementation of the developmental phenotypes of a lipid biosynthetic mutant upon exogenous lipid treatment (step 2). This problem might arise if the lipid is not stable in solution or if it has a high turnover rate *in vivo*.

### Potential solution

Treat the plants regularly with a fresh lipid solution. If the plants are growing on plates, regularly transplant the seedling on new plates containing media with freshly added lipids. Alternatively, fresh liquid ½ MS with lyso-lipid can be added regularly on the plant surface using a pipette (Poulsen et al., 2015). If plants are growing on soil it may be possible to spray or water the plants with a freshly prepared lipid solution.

### Problem 5

The root samples have been damaged during the slide mounting process (step 4).

### Potential solution

This problem occurs when accidentally squeezing the sample by putting the coverslip. The morphology of the root tip should be checked under transmitted light and the cells should be fully turgescent (the plasma membrane is aligned on the cell wall on not retracted, which can be seen using the fluorescence of the PS sensor, but also under the transmitted light). In that case: 1) discard the damaged root and observed the other root mounted on the same slide, 2) mount new samples and make sure to be gentle when placing the coverslip, and 3) use spacers between the slide and coverslip (see the optional step for mounting the sample).

## Resource availability

### Lead contact

Further information and requests for resources and reagents should be directed to and will be fulfilled by the lead contact, Yvon Jaillais (yvon.jaillais@ens-lyon.fr).

### Materials availability

This study did not generate new unique reagents.

### Data and code availability

This study generated a code related to the macro to analyze the rosette size and can be found on github (https://github.com/mplatre/Macro_Rosette_Size/releases/tag/V1).
